# Metatranscriptomics and Amplicon Sequencing Reveal Mutualisms in Seagrass Microbiomes

**DOI:** 10.3389/fmicb.2018.00388

**Published:** 2018-03-15

**Authors:** Byron C. Crump, John M. Wojahn, Fiona Tomas, Ryan S. Mueller

**Affiliations:** ^1^College of Earth, Ocean, and Atmospheric Sciences, Oregon State University, Corvallis, OR, United States; ^2^Department of Fisheries and Wildlife, Oregon State University, Corvallis, OR, United States; ^3^Instituto Mediterráneo de Estudios Avanzados (IMEDEA), Universitat de les Illes Balears (UIB) – Consejo Superior de Investigaciones Científicas (CSIC), Esporles, Spain; ^4^Department of Microbiology, Oregon State University, Corvallis, OR, United States

**Keywords:** symbiosis, estuary, marine, microbiology, DNA, eelgrass, diazotroph, PCR

## Abstract

Terrestrial plants benefit from many well-understood mutualistic relationships with root- and leaf-associated microbiomes, but relatively little is known about these relationships for seagrass and other aquatic plants. We used 16S rRNA gene amplicon sequencing and metatranscriptomics to assess potential mutualisms between microorganisms and the seagrasses *Zostera marina* and *Zostera japonica* collected from mixed beds in Netarts Bay, OR, United States. The phylogenetic composition of leaf-, root-, and water column-associated bacterial communities were strikingly different, but these communities were not significantly different between plant species. Many taxa present on leaves were related to organisms capable of consuming the common plant metabolic waste product methanol, and of producing agarases, which can limit the growth of epiphytic algae. Taxa present on roots were related to organisms capable of oxidizing toxic sulfur compounds and of fixing nitrogen. Metatranscriptomic sequencing identified expression of genes involved in all of these microbial metabolic processes at levels greater than typical water column bacterioplankton, and also identified expression of genes involved in denitrification and in bacterial synthesis of the plant growth hormone indole-3-acetate. These results provide the first evidence using metatranscriptomics that seagrass microbiomes carry out a broad range of functions that may benefit their hosts, and imply that microbe–plant mutualisms support the health and growth of aquatic plants.

## Introduction

Bacteria and Archaea associated with plant leaves (phyllosphere microbiome) and roots (rhizosphere microbiome) can have positive impacts on the health of terrestrial plants (Kent and Triplett, 2002; Vorholt, 2012; Turner et al., 2013), but relatively little is known about how microbiomes impact the health of seagrass and other aquatic plants (Turner et al., 2013; Fahimipour et al., 2016). In terrestrial plants, these mutualistic functions include outcompeting pathogenic soil microbes, modulating plant immunity, fixing nitrogen for use by plants (Gruber and Galloway, 2008), and neutralizing harmful products (e.g., methanol and ethanol) exuded from leaves and roots (Abanda-Nkpwatt et al., 2006; Turner et al., 2013).

Terrestrial plant rhizosphere and phyllosphere microbiomes are generally composed of different organisms with different relationships to their host plant. Rhizosphere microbiomes are mainly derived from soil microbiota (Kent and Triplett, 2002) and influenced by chemicals exuded by the plant roots (Haichar et al., 2008). In contrast, terrestrial phyllosphere microbiome source communities are unclear (Vorholt, 2012), but their compositions are strongly influenced by abiotic environmental factors, such as precipitation and light exposure (Turner et al., 2013). Similar contrasts between rhizosphere and phyllosphere microbiomes likely exist for aquatic plants, but relationships with their hosts may involve different processes or use different mechanisms because aquatic plant leaves are often submerged in water, and roots are anchored in water-saturated sediments.

Seagrasses are aquatic flowering plants that form the base of productive coastal ecosystems and provide habitat for many marine organisms such as fish, shellfish, crabs, and algae (Boström et al., 2006). Seagrass beds contribute to important ecosystem services, including nutrient cycling (McGlathery et al., 2007), storm-surge damping (Spalding et al., 2014), water clarification, and by acting as a global carbon sink (Orth et al., 2006). Also, decomposition of eelgrass detritus fuels a variety of food webs, both local and distal to the beds (Hemminga and Duarte, 2000).

*Zostera marina* (eelgrass) is the most widespread species, present throughout the coasts of the North Atlantic and North Pacific oceans (Green and Short, 2003). In estuaries on the United States Pacific coast, seagrass beds are typically dominated by *Z. marina*, but, since 1957, an invasive seagrass from Japan, *Zostera japonica* (Japanese eelgrass), has become established (Baldwin and Lovvorn, 1994). *Z. japonica* has smaller, thinner leaves and rhizomes than *Z. marina* (Harrison, 1982). *Z. japonica* usually colonizes mid-to-low intertidal mudflats and *Z. marina* is dominant in low to subtidal areas, but both species often co-exist in some locations (Shafer et al., 2014) forming mixed beds that are useful for comparing microbiomes between plants and investigating potential microbiome mutualisms across plant species.

Currently, there are two well-described seagrass–microbe mutualistic relationships. First, sulfur-oxidizing bacteria in seagrass root microbiomes use oxygen provided by the plant roots (Pedersen et al., 2004; Jensen et al., 2007; Hasler-Sheetal and Holmer, 2015) to oxidize sulfide as part of their metabolism (acting alone or in cooperation with root-associated clams; van der Heide et al., 2012). Sulfide is toxic to seagrasses (Goodman et al., 1995; Koch, 2001; Baden et al., 2003) and, thus, the growth of sulfur-oxidizing organism on eelgrass root surfaces benefits seagrass by detoxifying sediments (Jensen et al., 2007; Crump and Koch, 2008; Fahimipour et al., 2016). Second, seagrass microbiomes fix nitrogen, which likely supports plant growth (Capone et al., 1979; Lipschultz et al., 1979; Capone, 1983; Pereg et al., 1994; Lehnen et al., 2016). Several studies have identified diverse nitrogenase genes (Bagwell et al., 2002; Lehnen et al., 2016) and 16S rRNA genes of potential diazotrophs (i.e., nitrogen fixing organisms) associated with eelgrass leaves and roots (Jensen et al., 2007; Crump and Koch, 2008).

There are also several other potential seagrass–microbe symbioses that are relatively well-known in land plants. For instance, land–plant microbiomes degrade a broad range of plant exudates and waste products (Dourado et al., 2013) including methanol (Jourand et al., 2005; Abanda-Nkpwatt et al., 2006) and ethanol (Williams and Yavitt, 2010). Plant microbiomes also produce and degrade many different plant hormones and growth regulators (Glick, 1995; Dodd et al., 2010), including auxins (e.g., indole-3-acetic acid; Patten and Glick, 1996; Omer et al., 2004), cytokinins (e.g., zeatin; Akiyoshi et al., 1984; Ivanova et al., 2000; Mok and Mok, 2001), ethylene (Glick et al., 1998; Meldau et al., 2012), and nitric oxide (Stohr and Ullrich, 2002). Plant microbiomes also control pathogens and other organisms that may cause harm to their host plants (Andrews, 1992; Whipps, 2001).

We investigated potential eelgrass–microbiome mutualisms in two co-occurring species of eelgrass (*Z. marina* and *Z. japonica*) using 16S rRNA gene amplicons sequencing and metatranscriptomics. We hypothesized that *Z. marina* and *Z. japonica* would share similar leaf and root microbiomes despite morphological differences between the plants. We tested this hypothesis by comparing the phylogenetic composition of leaf and root microbial communities between the two species and with bacterioplankton collected from nearby, but independent, sampling sites. Additionally, we used metatranscriptomic data from these microbiomes to investigate a broad range of potential mutualistic processes between seagrasses and microbes, including sulfur oxidation and nitrogen fixation. Potential mutualisms were hypothesized based on the analysis of relative gene-expression patterns between leaves and roots of the two plant species with metatranscriptomic sequencing, and comparison of these results with typical coastal bacterioplankton metatranscriptomes from a prior study of the nearby Columbia River estuary and river plume.

## Materials and Methods

### Sample Collection

Samples were collected during low tides on July 28 and September 8, 2014 from two shallow pools (∼10 cm) within tidal mudflats harboring mixed beds of *Z. marina* and *Z. japonica* in Netarts Bay, OR, United States (Latitude: 45.394139° and Longitude: -123.939172°). This is a marine-dominated, strongly tidally flushed estuary in which approximately 75% of the water in the bay is replaced with each tidal cycle (Glanzman et al., 1971).

**FIGURE 1:**
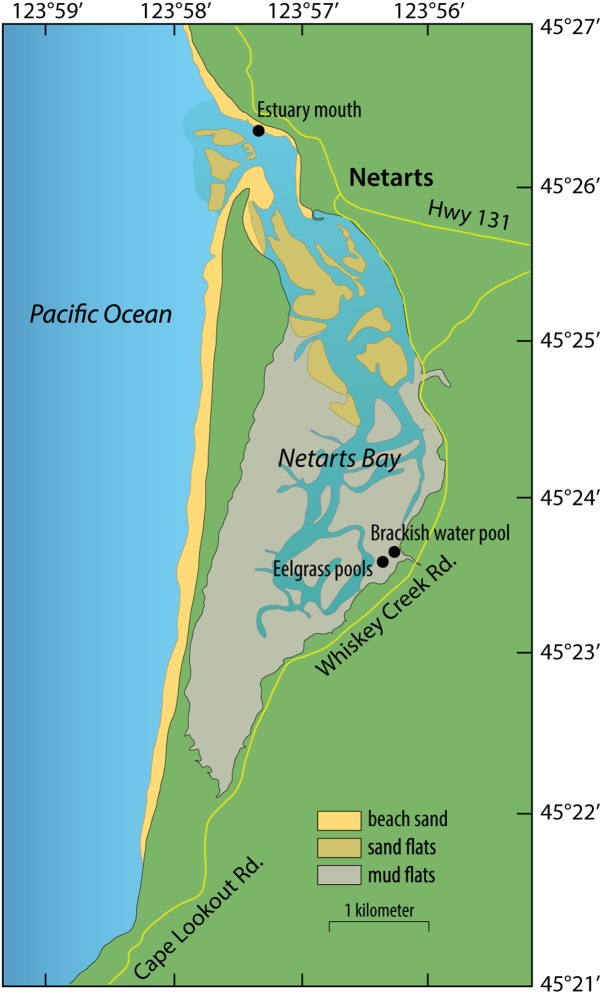
Map of sampling sites in Netarts Bay, OR, United States.

Whole plants were gently removed from sediment with gloved fingers and rinsed with filter-sterilized (Sterivex-GP 0.22 μm, EMD Millipore, Darmstadt, Germany) seawater. Roots and leaves were separated from rhizomes, placed in 50 ml sterile tubes, and rinsed 3 to 5 times with gentle inversion. Samples were fixed in RNAlater, placed on dry ice, and stored at -20°C until extraction. July samples were rinsed with filter-sterilized brackish water collected in a nearby tidal pool at the mouth of a small stream (Latitude: 45.394877° and Longitude: -123.938005°; **Figure 1**), and September samples were rinsed with filter-sterilized seawater collected from the mouth of the estuary (Latitude: 45.439343° and Longitude: -123.955221°; **Figure 1**). Microbes captured on the Sterivex filters used to prepare rinse water were preserved with 1 mL of filter-sterilized DNA extraction buffer [DEB; 0.1 M Tris-HCL (pH 8), 0.1 M Na-EDTA (pH 8), 1.5 M NaCl, 5% Cetyltrimethyl ammonium bromide], and stored at -80°C.

### Microbial Community Composition

DNA was extracted from leaf and root tissues (triplicate samples of leaf and root tissues from each plant species on each sampling date) and from filtered seawater samples to measure the phylogenetic composition of plant-associated and planktonic bacterial communities. Thawed root and leaf samples were soaked in sterile ultrapure water at 4°C for 20 min to remove RNAlater, and cut into small pieces. DNA was extracted with the PowerBiofilm DNA Isolation Kits (MO BIO Laboratories, Inc.) following manufacturer’s instructions. DNA from water-column microbes collected on Sterivex filters was isolated using a phenol–chloroform extraction method (Crump et al., 2003). Bacterial community composition was determined with PCR amplicon sequencing of 16S rRNA genes following Kozich et al. (2013), with dual-barcoded versions of the PCR primers from Caporaso et al. (2012). The V4 hypervariable region of 16S rRNA genes were PCR amplified with 250 nM primers (final concentration) and HotMasterMix (5 Prime) under the following conditions (94°C for 3 min; 30 cycles of 94°C for 45 s, 50°C for 60 s, 72°C for 90 s; 72°C for 10 min). Three technical PCR replicates were performed for each sample, pooled, and quantified using Picogreen. Amplicons were then pooled at equimolar concentrations, cleaned using a MoBio Ultraclean PCR Clean-Up Kit, quantified using Picogreen, and sequenced at the Oregon State University Center for Genome Research and Biocomputing (CGRB) using the Illumina MiSeq platform and v2 chemistry (2 × 251 base long, paired-end reads).

Amplicon sequences were paired using make.contigs from the ‘mothur’ package (v.1.32.1) (Schloss et al., 2009), converted to QIIME format with split.groups from ‘mothur’ and add_qiime_labels.py from the QIIME software package (Caporaso et al., 2010). Sequences were quality filtered with an expected error rate of 0.5, dereplicated (derep_fulllength), and abundance sorted (sortbysize) using USEARCH (v.7.0.1001_i86linux64) (Edgar, 2013). Singleton sequences were removed and reads were clustered into operational taxonomic units (OTUs) at 97% similarity (cluster_otus). A *de novo* chimera check is inherent in the cluster_otus algorithm, but a reference-based chimera filtering was also performed (uchime_ref) with the Gold Database^1^. All reads (including singletons) were subsequently mapped back to representative OTU sequences using UPARSE (usearch_global), and an OTU table listing relative abundances between samples was created. Taxonomy of the representative sequences was assigned in QIIME (assign_taxonomy.py) using the RDP classifier trained to the SILVA database (v.111 database clustered to 97% OTUs). Alpha-diversity was calculated using Catchall within ‘mothur,’ and calculations of similarity matrices, MDS diagrams, ANOSIM, and SIMPER analyses were carried out using PRIMER v6 (PRIMER-E Ltd., Plymouth, United Kingdom).

### Metatranscriptomics

Gene-expression patterns of microbial communities were assessed in four tissue samples (*Z. marina* leaf, Z. *japonica* leaf, Z. *marina* root, and Z. *japonica* root) using metatranscriptomic sequencing of mRNA. These sequences were compared to previously published metatranscriptomic sequences collected from planktonic microbes in the nearby Columbia River estuary and river plume (Fortunato and Crump, 2015). To extract RNA, thawed tissues were removed from RNAlater and cut into small pieces, and cells suspended in RNAlater were captured on Sterivex-GP 0.22-micron filters. The filter material was removed from the Sterivex-GP filter capsule, sliced into pieces, and combined with the tissue fragments. Total RNA was isolated with the MoBio PowerSoil Total RNA Isolation Kit (MO BIO Laboratories, Inc.) following manufacturer’s instructions, DNA was removed using the Ambion TURBO DNA-free kit, and rRNA was depleted with sequential use of two Ribo-Zero Gold kits (Bacterial rRNA, Plant rRNA; Illumina, Inc.) following Benes et al. (2011). Sequencing libraries made from rRNA-depleted samples were constructed with a Wafergen Apollo 324 robot using the PrepX RNA-Seq for Illumina library prep kit. Sequencing was carried out on an Illumina HiSeq 3000 sequencer using paired-end 150 base long reads at CGRB (total 8.4 GB). Metatranscriptome sequence data was deposited with links to BioProject accession number PRJNA419030 in the NCBI BioProject database under accession numbers SRR6310506-SRR6310509^2^.

Metatranscriptomes from the water column of the Columbia River estuary and river plume (Fortunato and Crump, 2015) were downloaded from the European Nucleotide Archive (accessions ERS709858 to ERS709862), quality controlled as above, mapped to the published contigs downloaded from the Integrated Microbial Genomes (IMGs) database (GOLD study ID Gs0084963). These five metatranscriptomes were from 2.0 μm pre-filtered water collected at salinities 0, 5, 15, 25, and 33 PSU in August 2010. RNA was extracted with the RNeasy kit (Qiagen) following Poretsky et al. (2009), rRNA depleted with subtractive hybridization (Stewart et al., 2010), and sequenced as 100 base long paired-end reads on an Illumina HiSeq 1000 system.

Metatranscriptome sequences were co-assembled with Megahit (Li et al., 2015) and CDS sequences were identified and annotated through the Microbial Genome Annotation Pipeline of the IMG online system (Chen et al., 2016). Raw paired-end sequences from each sample were quality controlled (trimfq command in seqtk v.1.0-r72-dirty, default settings) and mapped to CDS sequences with Bowtie2. CDS sequences assigned to eukaryotes and viruses were excluded from analysis. CDS sequences assigned to Cyanobacteria and Fusobacteria included a large proportion of photosynthesis gene transcripts, and so were also excluded because it was unclear whether these were mis-assigned chloroplast transcripts. Abundances of transcripts that mapped to KEGG-annotated CDS sequences were normalized as transcripts per million (TPM) following Wagner (Wagner et al., 2012) to account for variations in sequence length and template length, and were analyzed using tools in MEGAN V.5 (Huson et al., 2011).

DNA sequences from this study are available from NCBI under accession numbers SRP125305 (Amplicon sequences) and SRP125305 (metatranscriptomes). Metatranscriptome assembled contigs and annotations are available from IMG/M ER^3^ under Taxon ID 3300008055.

## Results

**FIGURE 2:**
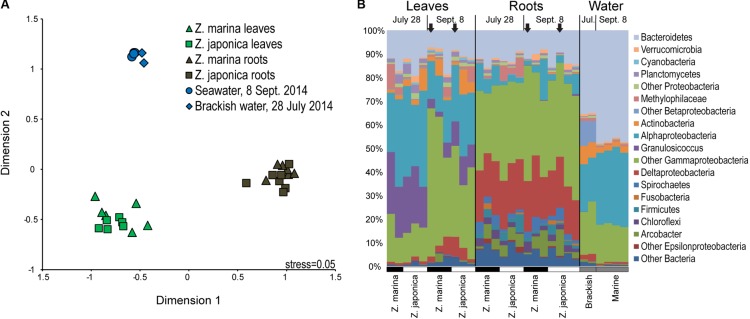
Multidimensional scaling diagram **(A)** showing beta-diversity of plant-associated and water column bacterial communities based on Bray–Curtis similarity calculated from the relative abundances of 16S rRNA gene amplicon sequences grouped into operational taxonomic units (OTUs) based on 97% DNA sequences similarity. **(B)** Taxonomic diversity of plant-associated and water-column bacterial communities based on 16S rRNA gene amplicon sequences grouped by major taxa with samples labeled by tissue type, date, and plant species. The black arrows indicate samples used for metatranscriptomics.

Seagrass-associated bacterial community composition was not significantly different between plant species for leaf (ANOSIM, *P* < 0.199) or root (ANOSIM, *P* < 0.091) microbiomes (**Figure 2A**). On the other hand, leaf, root, and water column communities were significantly different from one another (ANOSIM, *P* < 0.001) (**Figure 2A**). Leaf microbiomes (**Figure 2B**) were dominated by Bacteroidetes, Alphaproteobacteria, and Gammaproteobacteria, the last of which included a large proportion of *Granulosicoccus* representing on average 13% of phyllosphere communities. Root microbiomes were dominated by Bacteroidetes, Deltaproteobacteria, and Gammaproteobacteria, with smaller proportions of Spirochaetes, Firmicutes, and the Epsilonproteobacteria genus *Arcobacter*. Bacterioplankton communities in water samples were dominated by Bacteroidetes, Alphaproteobacteria, and Gammaproteobacteria, with elevated proportions of Actinobacteria and Betaproteobacteria in brackish water samples from July (**Figure 2B**).

SIMPER analysis identified indicator taxa differentiating eelgrass leaf communities from root communities (Supplemental Table S1). Leaf indicators were from the Alphaproteobacteria family Rhodobacteraceae, the Betaproteobacteria family Methylophilaceae, the Bacteroidetes genus *Polaribacter*, and the Gammaproteobacteria genera *Granulosicoccus*, *Simiduia*, and *Marinomonas*. Also, one dominant indicator classified to class Gammaproteobacteria (OTU 69, 3.2% of leaf microbiome), was 93% similar to the methylotrophic organism *Methylobacter marinus*. Root indictors were classified to the Epsilonproteobacteria genus *Arcobacter*, the Betaproteobacteria genus *Methylotenera*, the Bacteroietes family *Marinilabiaceae*, the Deltaproteobacteria families Desulfobacteraceae and Desulfobulbaceae, and the Gammaproteobacteria genera *Sedimenticola*, *Reinekea*, and *Vibrio.* Root indicators also included three very abundant OTUs (>1% of root microbiome) that were only classified to class Gammaproteobacteria: OTU 6 (11.6% of root microbiome) was 100% similar to *Spongiibacter marinus* DSM 19753 (GI: 523385909); OTU 30 (3.0% of root microbiome), was 92% similar to the sulfur-oxidizer *Thiomicrospira chilensis* DSM 12352; and OTU 75 (1.6% of root microbiome) was 98% similar to sulfur oxidizing symbionts from *Ridgeia piscesae* (GI: 410699265) and *Riftia pachyptila* (GI: 28913259).

Co-assembly of 5.6 million metatranscriptome sequence reads (**Table 1**) produced 1.8 million contigs (N50 = 1,058) containing 2.4 million CDS (median length 315 bp). For the metatranscriptome sequences collected from each sample, 13–37% of sequences were assigned to CDS annotated to KEGG functions, of which 15–81% were assigned to Eukaryotes, and 9–80% (0.5–2.1 million reads) were assigned to Bacteria and Archaea other than Cyanobacteria and Fusobacteria (**Table 2**). Recovery of seagrass microbiome mRNA sequences was similar to other plant microbiome metatranscriptome studies (Cao et al., 2015; Marzano and Domier, 2016) in which a large percentage of mRNA sequences were derived from the host plant.

**Table 1:** Megahit co-assembly statistics for metatranscriptomes sequences.

	Megahit co-assembly
Total sequence reads	55,700,636
Reads mapped to contigs	41,258,794
Number of contigs	1,836,640
Min contig length	500
Max contig length	112,917
Mean contig length	1029
Median contig length	738
N50 contig length	1058
Number of CDS	2,358,557


**Table 2:** Metatranscriptome mapping and annotation results from eelgrass microbiomes.

	*Z. marina* leaf	*Z. marina* root	*Z. japonica* leaf	*Z. japonica* root
Total pairs of reads	8,909,964	6,709,217	4,717,817	7,513,320
Total number of reads	17,819,928	13,418,434	9,435,634	15,026,640
Reads mapped to CDS	15,081,961	8,393,145	7,631,476	10,152,212
KEGG annotated reads	6,569,893	1,774,463	1,812,490	2,722,803
Bacteria and Archaea^∗^	613,963	1,415,784	504,781	2,124,317
Bacteria	1,211,293	1,495,747	725,098	2,195,695
Eukaryotic	5,353,704	274,622	1,085,762	520,245
Archaea	3,163	3,245	1,008	6,585
Virus	1,733	849	622	278


*^∗^Not including Cyanobacteria or Fusobacteria.*

For the metatranscriptome sequences collected from the nearby Columbia River estuary (Fortunato and Crump, 2015), 1–11% mapped to KEGG-annotated CDS, of which 1–7% were assigned to Eukaryotes, and 90–99% (1.1–10.3 million reads) mapped to Bacteria and Archaea other than Cyanobacteria and Fusobacteria (**Table 3**).

**Table 3:** Metatranscriptome mapping and annotation results from the water column of the Columbia River estuary.

	Columbia River (0 PSU)	Columbia River estuary (5 PSU)	Columbia River estuary (15 PSU)	Columbia River plume (25 PSU)	Coastal water (33 PSU)
Total pairs of reads	63,587,948	50,795,108	50,717,926	39,388,894	22,118,990
Total number of reads	127,175,896	101,590,216	101,435,852	78,777,788	44,237,980
Reads mapped to CDS	30,093,940	75,092,069	32875061	50,916,873	18,716,051
KEGG annotated reads	1,135,795	11,534,766	1,159,297	7,227,746	2,211,849
Bacteria and Archaea^∗^	1,089,599	10,338,180	1,121,236	6,828,779	2,181,288
Bacteria	1,089,968	10,554,460	1,121,722	6,876,722	2,176,794
Eukaryotic	43,934	848,597	34,499	287,638	25,231
Archaea	1,893	128,580	2,728	59,985	9,133
Virus	0	3,129	348	3,401	691


*^∗^Not including Cyanobacteria or Fusobacteria.*

**FIGURE 3:**
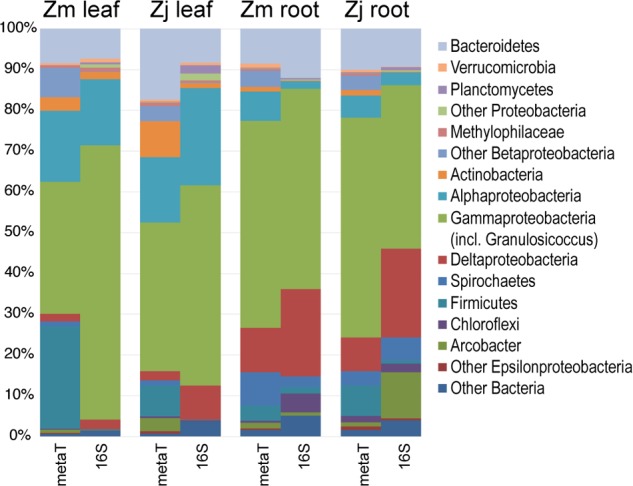
Comparison of the taxonomy of KEGG-annotated genes from metatranscriptomes with 16S rRNA gene amplicon sequences. 16S genes assigned to the genus *Granulosicoccus* were grouped with Gammaproteobacteria. Transcripts and 16S genes assigned to Cyanobacteria and Fusobacteria were omitted. (Zm: *Z. marina*; Zj: *Z. japonica*)

**FIGURE 4:**
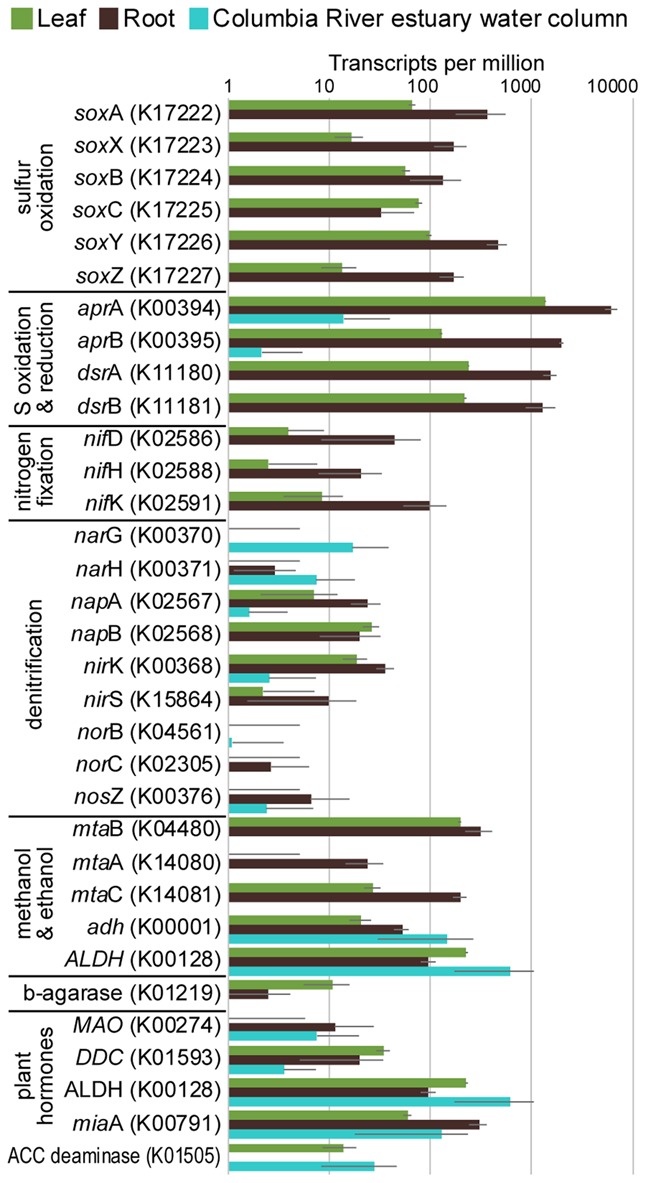
Average transcripts per million (TPM) for select genes from metatranscriptomes of eelgrass leaves and roots, and from the water column of the Columbia River estuary. Error bars indicate standard deviation.

**FIGURE 5:**
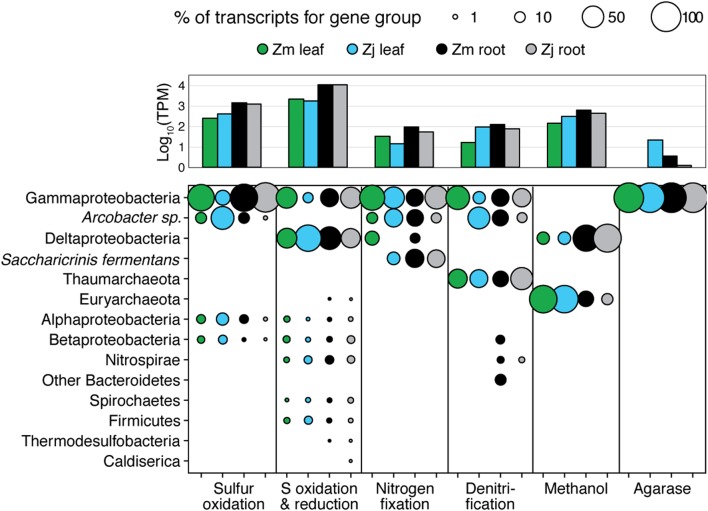
Taxonomy of gene transcripts for gene sets from **Figure 3**. Column graph shows the sums of relative expression (log_10_ of transcripts per million) of genes in each category for each sample type. Bubble size indicates percent of TPM for each sample within each gene set.

The taxonomic composition of microbial communities based on metratranscriptome sequences was similar to that based on 16S gene amplicon sequences (**Figure 3**). Transcripts were detected for genes encoding functions potentially involved in plant–microbe mutualisms, including sulfur oxidation, nitrogen fixation, denitrification, methanol and ethanol consumption, beta-agarase production, and plant hormone synthesis (**Figure 4**). Sulfur oxidation genes on roots and leaves (*soxABCXYZ*) were expressed by *Arcobacter* sp., and genes involved in sulfur oxidation and reduction (*aprAB*, *dsrAB*) were expressed by both sulfur-oxidizing Gammaproteobacteria and sulfate-reducing Deltaproteobacteria (**Figure 5**). Nitrogen fixation genes (*nifHDK*) were primarily expressed by sulfur-oxidizing Gammaproteobacteria, *Arcobacter* sp., and a known nitrogen-fixing Bacteroidetes. Methanol consumption genes (*mtaABC*) were expressed primarily by Deltaproteobacteria on roots and by Euryarchaeota on leaves. Denitrification genes were expressed by Gammaproteobacteria, *Arcobacter* sp., and Thaumarchaeota, and beta-agarase was expressed by Gammaproteobacteria.

## Discussion

The recent expansion of microbiome research demonstrates the close relationships between microbes and their hosts including terrestrial plants (Berendsen et al., 2012), insects (Husnik et al., 2013), and human beings (Huttenhower et al., 2012). Microbiomes of terrestrial plants have been extensively studied (e.g., Kloepper et al., 1980), but microbiomes of aquatic plants have only recently begun to be explored (Uku et al., 2007; Crump and Koch, 2008; Gordon-Bradley et al., 2014; Cucio et al., 2016; Mejia et al., 2016; Ettinger et al., 2017). Several of these studies show that the composition of these microbiomes differs from those in sediment and the water column, but no studies have used metatranscriptomics to describe how these microbiomes interact with their host plants.

In addition to the major physical and chemical controls of seagrass production, such as light and nutrient availability (Touchette and Burkholder, 2000; Ralph et al., 2007), associated microbiomes may also influence plant productivity and health by performing mutualistic functions. Our metatranscriptome results, which captured a snapshot of microbiome gene expression during the day at low tide, suggested that eelgrass microbiomes detoxify compounds that inhibit plant growth (e.g., sulfide, methanol, ethanol), fix nitrogen, and produce agarases that may cause disease and die-off in competitive algal epibionts. These metatranscriptomic results also suggest that eelgrass microbiomes carry out all of the steps involved in denitrification, and in the production of the plant-growth hormone indole-3-acetate (IAA).

The beta-diversity of Z. *marina* and *Z. japonica* microbiomes was consistent with several earlier seagrass studies showing many differences between leaf and root communities (Crump and Koch, 2008; Fahimipour et al., 2016; Ettinger et al., 2017), but few discernable differences in the microbiomes of different plant species collected at the same time and location (Cucio et al., 2016). Some earlier studies disagree with this second result and suggest that microbiomes vary with host plant species. However, these studies sampled plants with different structure or physical characteristics (He et al., 2012; Gordon-Bradley et al., 2014), or sampled plants from separate monospecific stands (Uku et al., 2007), or during different times of the year (Crump and Koch, 2008), all of which may influence microbiome composition. Cucio et al. (2016) sampled several co-occurring seagrass species, and found no differences in rhizosphere microbial communities in particles rinsed off roots despite the fact that each species was rooted in different types of sediment within the same habitat. These results suggest that seagrass microbiomes are not co-evolved with their hosts or controlled by sediment type, but instead are controlled by general seagrass metabolic processes (Cucio et al., 2016).

We found large differences between leaf microbiomes and water-column communities, which is inconsistent with a recent global study of eelgrass microbiomes (Fahimipour et al., 2016). In this global study, *Z. marina* leaf microbiomes were similar to local bacterioplankton, and a source-tracking analysis suggested leaf microbiomes were inoculated from the water column. Water samples for this global study were collected above submerged eelgrass beds, potentially capturing organisms dislodged from leaves. Also, leaf samples were not rinsed with sterilized water, which may have retained water column microbes in leaf samples. In contrast, our sampling approach aimed at limiting cross-contamination of leaf and water communities by washing leaf surfaces with filter-sterilized seawater, and by collecting water samples from nearby, but independent, sites. Differences in these two studies suggests that there is a dynamic exchange between eelgrass leaf microbiomes and the water column when plants are submerged, and that leaf microbiomes may be significant sources of organisms to local bacterioplankton.

The dominant taxa associated with leaves and roots generally coincided with earlier studies of seagrass microbiomes, but with several notable exceptions. For leaf microbiomes, Ettinger et al. (2017) found higher proportions of typically anaerobic Clostridiales in *Z. marina* leaf microbiomes from Bodega Harbor in California (included in the Firmicutes group in **Figure 2**), and Mejia et al. (2016) and Weidner et al. (2000) found lower proportions of Bacteroidetes and Gammaproteobacteria associated with the tropical seagrass *Halophila stipulacea* in the highly saline Gulf of Elat (Aqaba). For root microbiomes, Ettinger et al. (2017) found higher proportions of Epsilonproteobacteria (average ∼18%), but Jensen et al. (2007) found these bacteria in proportions similar to our study (1–12%). Differences among these studies may be driven by differences in regional environmental conditions, but could also be caused by differences in sampling techniques.

Seagrass microbiome studies have used DNA extracted from un-rinsed tissues (Fahimipour et al., 2016; Ettinger et al., 2017), from tissues rinsed with sterile water (this study; Jensen et al., 2007; Crump and Koch, 2008; Jiang et al., 2015), from sediment washed off of tissues (Cucio et al., 2016), and from material scraped or sonicated off the surfaces of un-rinsed and rinsed tissues (Weidner et al., 1996, 2000; Mejia et al., 2016; Bengtsson et al., 2017). These methodological differences limit our ability to compare results among studies, and favor the unified approach of Fahimipour et al. (2016). We extracted DNA from rinsed tissues in order to target the plant microbiomes that were firmly attached to tissues, and we found that the taxonomy and gene expression of these organisms provided information about known and novel interactions between seagrasses and their microbiomes.

### Sulfide Detoxification

Sulfide is highly toxic to eelgrass photosynthetic pathways (Goodman et al., 1995), and high sediment sulfide concentrations can lead to a decrease in the ATP available to plant cells (Koch, 2001). Oxidation of toxic sulfide by sulfur-oxidizing bacteria within the plant rhizosphere is a well-known mutualism in seagrasses, which transport oxygen to their roots via aerenchyma to limit tissue anaerobiosis (Hasler-Sheetal and Holmer, 2015), and, in turn, provide oxygen for use as the terminal electron acceptor by sulfur-oxidizing bacteria (van der Heide et al., 2012). We identified several sulfur-oxidizing taxa on roots (Flood et al., 2015; Han and Perner, 2015; Hansen and Perner, 2015), including members of the genera *Sedimenticola*, *Arcobacter*, *Thiomicrospira*, and *Sulfuromonas*. These taxa averaged 11% of root microbiomes (primarily the root indicator taxa *Sedimenticola* and *Arcobacter*) and less than 2% of leaf and water column microbiomes. Similarly, typical sulfate-reducing taxa belonging to the families Desulfobacteraceae, Desulfobulbaceae, Desulfuromonadaceae, and Desulfovibrionaceae accounted for 16% of root microbiomes but less than 2% of leaf and water-column microbiomes, confirming the importance of sulfur cycling in eelgrass rhizosphere (Pedersen et al., 2004; Fahimipour et al., 2016).

Metatranscriptomic analysis identified expression of many genes involved in sulfur oxidation including a sulfur-oxidizing enzyme complex (*soxABCXYZ*) used by many chemo- and photolithoautotrophic sulfur-oxidizing organisms (Friedrich et al., 2005). In root microbiomes, these genes were almost exclusively assigned to several Gammaproteobacteria genera including *Sedimenticola*, *Thiohalomonas*, *Dechloromarinus*, an endosymbiont of *Riftia pachyptila*, and to the Epsilonproteobacteria *Arcobacter nitrofigilis* (**Figure 5**).

We also identified expression of genes involved in both the oxidation of sulfide and reduction of sulfate, including dissimilatory sulfite reductase genes *dsrA* and *dsrB*, and adenylyl-sulfate reductase genes *aprA* and *aprB* (Watanabe et al., 2016). These genes were assigned to both sulfur-oxidizing Gammaproteobacteria (e.g., *Sedimenticola*, *Thiohalomonas*, *Dechloromarinus*) and sulfate-reducing Deltaproteobacteria (e.g., *Desulfococcus*, *Desulfospira*, *Desulfopila*, *Desulfobacula*). Expression of these genes was always much greater for root microbiomes than leaf or water column microbiomes (**Figure 4**), and accounted for >1% of transcripts from root samples. These results suggest that a large and diverse portion of the eelgrass root microbiome is dedicated to sulfur oxidation, which likely benefits both microbes and plants.

### Nitrogen Fixation and Denitrification

Rates of nitrogen fixation by seagrass leaf and root microbiomes were first published in Goering and Parker (1972) and Patriquin and Knowles (1972), and although these rates are sometimes highly variable (Capone et al., 1979; Lehnen et al., 2016), they are thought to account for a significant portion of plant nitrogen demand (Lipschultz et al., 1979; Capone, 1983; Pereg et al., 1994). Nitrogen-fixing bacteria have been identified on seagrass leaves and roots using 16S rRNA gene sequencing (Jensen et al., 2007; Crump and Koch, 2008), and nitrogenase (*nifH*) gene sequencing (Bagwell et al., 2002; Lehnen et al., 2016). One study sequenced *nifH* genes in sediments of a tropical seagrass bed of *Thalassia testudinum* and *Syringodium filiforme* and identified genes assigned to several classes of Proteobacteria (Bagwell et al., 2002). More recently, *nifH* sequences from *Posidonia oceanica* leaves, roots, and rhizomes confirmed that a high diversity of nitrogen-fixing bacteria is associated with plant surfaces, with many of these sequences being assigned to sulfate-reducing Deltaproteobacteria (Lehnen et al., 2016).

Our 16S sequencing identified a high proportion of organisms related to *Arcobacter nitrofigilis* (1% of leaf and 4% of root), which is a nitrogen-fixing symbiont on the roots of the marsh grass *Spartina alterniflora* (Pati et al., 2010). Expression of *nifD*, *nifE*, and *nifH* genes was much higher on roots than on leaves or in the water column, likely reflecting the oxygen sensitivity of nitrogenase (**Figure 4**; Gallon, 1992). Some of these transcripts were assigned to *Arcobacter nitrofigilis*, but most were assigned to sulfur-oxidizing Gammaproteobacteria and to the nitrogen-fixing Bacteroidetes, *Saccharicrinis fermentans* (Inoue et al., 2015). In contrast, very few nitrogenase transcripts were assigned to sulfate-reducing Deltaproteobacteria (**Figure 5**) suggesting that, despite their relative abundance, these organisms are not the main nitrogen fixers in the seagrass microbiome.

Microbially-mediated denitrification is an important ecosystem service of estuarine seagrass beds (Reynolds et al., 2016), which acts to remove excess nitrogen from eutrophic systems, and may influence plant growth through the generation of nitric oxide (NO; Stohr and Ullrich, 2002), an important regulator of development in plants (Domingos et al., 2015). Denitrification rates are variable in seagrass beds (Risgaard-Petersen and Ottosen, 2000; Eyre et al., 2011; Piehler and Smyth, 2011), and often lower than nitrogen fixation rates (Welsh et al., 2000; Russell et al., 2016). We detected higher expression of most denitrification genes in root versus leaf metatranscriptomes (**Figure 4**) with the exception of the *norB* gene coding for part of the NO reductase complex, suggesting that NO may be an important end product of denitrification in root microbiomes. Transcripts for each gene mapped to different organisms (e.g., *nirK* to Thaumarchaeota, *nirS* to Gammaproteobacteria and Betaproteobacteria, *napB* to *Arcobacteria* sp., *nosZ* to Bacteroidetes; Supplementary Table S2), suggesting that denitrification on plant roots involves the cooperation of several different denitrifying species.

### Microbial Consumption of Plant Exudates

Angiosperms produce methanol as a by-product of cell-wall synthesis (Nemecek-Marshall et al., 1995), yet this same product can inhibit germination and retard the growth of angiosperm seedlings (Abanda-Nkpwatt et al., 2006). In strawberry plants, this negative effect is mitigated by methanol-consuming *Methylobacterium extorquens* (Abanda-Nkpwatt et al., 2006; Kurilenko et al., 2010). Methanol-consuming bacteria are common in marine environments, and include members of the genera *Methylophaga* and *Methylobacter.*

The family Methylophilaceae averaged 7% of the leaf microbiome and 4% of the root microbiome in our samples, compared to 1% in water samples, and belonged to four OTUs (**Figure 2**). However, we detected almost no expression of the methanol dehydrogenase genes (*mdh* and *mxaIF*) that are commonly involved in methanol oxidation. Instead, our metatranscriptomic analysis found expression of the genes *mtaABC* by leaf and root microbiomes (**Figure 4**). These genes code for a protein complex that irreversibly transforms methanol into methyl-CoM, which is the first step in the disproportionation of methanol to CO_2_ and methane (Sauer and Thauer, 1999). These genes were expressed mainly by Euryarchaea on leaves, and by Deltaproteobacteria on roots (**Figure 5**). Expression was also detected for heterodisulfide reductase genes (*hdrABC* and *mvhADG*), but expression was very low or absent for other genes involved in methane production including methyl-coenzyme M reductase genes (*mcrABCDG*) and tetrahydromethanopterin *S*-methyltransferase genes (*mtrABCDEFGH*), suggesting that methane is not produced by the eelgrass microbiome.

Terrestrial plants release methanol in large quantities, rivaling the release of other volatile organic compounds (monoterpenes and isoprene), but they release much of this material via stomata to the atmosphere (Nemecek-Marshall et al., 1995). Seagrasses do not have stomata (Kuo and den Hartog, 2007), and likely release plant-produced methanol and other volatile organics via diffusion through root tissue and through their thin leaf cuticle.

Ethanol and acetaldehyde are produced by terrestrial plant roots when soils become anoxic following flooding, causing plants to switch from an aerobic to a fermentative metabolism (Kreuzwieser et al., 1999). Release of these compounds generally follows a diurnal pattern, with no release at night and a burst in the morning when stomata open (Rottenberger et al., 2008). Seagrasses are often rooted in anoxic sediments and use fermentation at night when their photosynthetic oxygen pool is depleted (Touchette and Burkholder, 2000; Pedersen et al., 2004). Under anoxic conditions *Z. marina* roots can produce ethanol, lactate, and other metabolites, and may release over 95% of the ethanol as exudate (Smith et al., 1988). Ethanol is thought to be toxic to plants only at high concentrations (Tadege et al., 1999), and its oxidation product acetaldehyde is considered highly toxic (Perata and Alpi, 1991). Exacerbating this stress is the fact that these compounds are released into anoxic sediments where they may be used as carbon sources by sulfate-reducing bacteria to generate toxic levels of sulfide (Widdel, 1988).

Many microbes are capable of metabolizing ethanol and acetaldehyde, and we found expression of alcohol dehydrogenase (*adh*) and aldehyde dehydrogenase (*ALDH*) genes in our samples (**Figure 4**). Each of these enzymes can broadly act on a range of alcohols and aldehydes as substrates, indicating that expression here may not be exclusively linked to ethanol and acetaldehyde metabolism. These genes were expressed by a broad range of taxa including Alpha-, Beta-, Gamma- and Delta-proteobacteria, Firmicutes, and Actinobacteria. Low expression of alcohol consumption genes is consistent with low daytime production of ethanol and acetaldehyde during our sampling, and thus, expression of these genes may increase at night when roots undergo anaerobiosis.

### Control of Epiphyte Community

As eelgrass leaves age, they accumulate epibiotic algal biofilms that can compete with the eelgrass leaves for light (Wahl, 1989). Epibiotic bacteria have been shown to influence the composition of biofilms on marine macroalgae, and, in doing so, provide protection to their hosts against extensive biofouling (Armstrong et al., 2001; Rao et al., 2005). One study showed that early colonization of *P. oceanica* seedlings by the bacteria *Marinomonas posidonica* influenced the composition of the epiphyte community and significantly increased leaf growth (Celdran et al., 2012). Another study showed that *Z. marina* leaves host high densities of algicidal bacteria (Inaba et al., 2017). One way for bacteria to influence algal growth is through the production of agarases and carrageenases that degrade galactose-based algal polymers and, in the case of agarases, can cause disease and die-off of red seaweed (Schroeder et al., 2003). We found that leaf microbiomes included a high proportion (average 3.3% leaf and 1.8% root) of organisms belonging to the Gammaproteobacteria genus *Simiduia*, which is associated with agarose hydrolysis (Park et al., 2014; Tawara et al., 2015). We also found expression of beta-agarase genes and several galactosidases were assigned to Gammaproteobacteria in the *Z. japonica* leaf microbiome (**Figure 5**). These organisms may be involved in regulating epiphyte communities, and, thus, could have important implications for epiphyte–seagrass competitive interactions.

### Plant Hormone Production

Auxins, such as IAA, are key regulators of plant growth and development. A binding site for an auxin response factor was detected in the *Z. marina* genome (Olsen et al., 2016), suggesting that this class of hormones might be used by seagrasses. However, one study of *Posidonia australis* found mixed effects of auxins on seedling survival (Glasby et al., 2015), and studies of *Halophila decipiens* and *Cymodocea nodosa* found no effect of auxin exposure on growth (Munoz, 1995; Bird et al., 1998).

We detected expression of three genes involved in bacterial conversion of tryptophan to IAA: *MAO*, which codes for tryptophan dehydrogenase (tryptophan to tryptamine; Shih et al., 1999), *DDC*, which codes for tryptamine oxidase (tryptamine to indole-3-acetaldehyde; Chassande et al., 1994), and *ALDH*, which is a family of enzymes that includes IAA dehydrogenases (indole-3-acetaldehyde to IAA; Basse et al., 1996). However, we did not find expression of the KEGG enzyme classified as IAA dehydrogenase (K11817), or of several genes involved in the more common indole-3-acetamide (Amin et al., 2015) and indole-3-pyruvate pathways for IAA production (Spaepen et al., 2007). Expression of a complete tryptamine pathway for IAA production suggests that seagrass microbiomes produce IAA and potentially influence plant growth through regulation of this compound.

Expression of genetic pathways for other plant hormones were limited and incomplete. For example, the cytokinin zeatin (Takei et al., 2004; Dodd et al., 2010) is present in the leaves and roots of the seagrass *P. oceanica* (L.) Delile, and shows a dynamic distribution in shoot tissues in relation to environmental stress factors (Bruno et al., 2009). Cytokinin is also produced by some bacteria using the *ipt* gene for cytokinin synthase (Mok and Mok, 2001). However, expression of this gene and all others in the zeatin biosynthesis pathway were not detected except the *miaA* gene coding for tRNA dimethylallyltransferase.

Another example of a plant signaling molecule is ethylene, which is a growth regulator produced by soil bacteria and plants (Zechmeister-Boltenstern and Smith, 1998; Hayat et al., 2010). Genomic analysis of the seagrasses *Z. marina* (Olsen et al., 2016) and *Z. muelleri* (Golicz et al., 2015) showed that genes for ethylene biosynthesis and signaling are missing from these genomes, suggesting that there is no need for microbes to participate in the regulation of ethylene levels. Consistent with this finding, no expression was detected of prokaryotic *efe* genes (K21815) that act in the synthesis of ethylene (Fukuda et al., 1992; Chen et al., 2010), and expression of ACC deaminase genes (K01505) for regulating plant synthesis of ethylene (Glick et al., 2007) was limited to *Z. marina* leaf and was lower than in bacterioplankton in the Columbia River estuary. These results suggest that seagrass microbiomes do not produce cytokinins or manipulate ethylene levels in *Z. marina* or *Z. japonica*.

## Conclusion

The phylogenetic composition of plant-associated bacterial communities was not significantly different between seagrass species for leaf microbiomes or root microbiomes. However, leaf-, root-, and water, column-associated bacterial communities were significantly different from one another. The taxonomy and gene expression of these microbiomes suggest that these communities detoxify sulfide using multiple metabolic pathways (e.g., *soxABCXYZ* and *dsrAB*), fix nitrogen, metabolize methanol and ethanol potentially released by eelgrass as waste products, produce agarases that may limit growth of competitive algal epiphytes, and influence plant growth by producing nitric oxide and the hormone IAA.

## Author Contributions

BC: project leadership, intellectual contributions, data analysis, and manuscript preparation. JW: laboratory analysis, intellectual contributions, data analysis, and manuscript preparation. FT: intellectual contributions and manuscript preparation. RM: intellectual contributions, data analysis, and manuscript preparation.

## Conflict of Interest Statement

The authors declare that the research was conducted in the absence of any commercial or financial relationships that could be construed as a potential conflict of interest.

## Footnotes

1 http://www.genomesonline.org/

2 https://www.ncbi.nlm.nih.gov/bioproject

3 http://img.jgi.doe.gov/mer
