# Supplementary material for: Metatranscriptomics and Amplicon Sequencing Reveal Mutualisms in Seagrass Microbiomes
Source: Front Microbiol. 2018 Mar 15;9:388. doi: 10.3389/fmicb.2018.00388 (PMC5863793; doi:10.3389/fmicb.2018.00388)
Supplement: Supplementary file 1 [file Table_1.DOCX]

Table S1. SIMPER results showing average relative abundances in leaf and root microbiomes, average dissimilarity (Av.Diss), quotient of dissimilarity and standard deviation (Diss/SD), contribution to differences between groups (Contrib%), and taxonomy of OTUs that contributed >0.7% to the difference between leaf and root microbiomes.

| Sample group | OTU# | Root Avg. Abund | Leaf Avg. Abund | Av.Diss | Diss/SD | Contrib% | Taxonomy |
| --- | --- | --- | --- | --- | --- | --- | --- |
| leaves | 15 | 0.1% | 13.8% | 6.86 | 1.70 | 7.3% | Bacteria; Proteobacteria; Gammaproteobacteria; Chromatiales; Granulosicoccaceae; Granulosicoccus |
| leaves | 91 | 0.1% | 4.0% | 1.96 | 1.47 | 2.1% | Bacteria; Proteobacteria; Alphaproteobacteria; Rhodobacterales; Rhodobacteraceae |
| leaves | 23 | 0.0% | 3.9% | 1.93 | 0.36 | 2.1% | Bacteria; Bacteroidetes; Flavobacteria; Flavobacteriales; Flavobacteriaceae; Polaribacter |
| leaves | 69 | 0.1% | 3.2% | 1.57 | 0.65 | 1.7% | Bacteria; Proteobacteria; Gammaproteobacteria |
| leaves | 27 | 0.0% | 3.0% | 1.48 | 0.91 | 1.6% | Bacteria; Actinobacteria; Acidimicrobiia; Acidimicrobiales; uncultured |
| leaves | 22 | 0.1% | 2.6% | 1.25 | 0.89 | 1.3% | Bacteria; Proteobacteria; Betaproteobacteria; Methylophilales; Methylophilaceae |
| leaves | 127 | 0.3% | 2.2% | 1.09 | 0.64 | 1.2% | Bacteria; Proteobacteria; Gammaproteobacteria; Alteromonadales; Alteromonadaceae; Simiduia |
| leaves | 77 | 0.0% | 2.2% | 1.08 | 0.79 | 1.2% | Bacteria; Proteobacteria; Alphaproteobacteria; Rhodobacterales; Rhodobacteraceae |
| leaves | 2664 | 0.1% | 2.0% | 0.99 | 0.50 | 1.1% | Bacteria; Proteobacteria; Gammaproteobacteria; Oceanospirillales; Oceanospirillaceae; Marinomonas |
| leaves | 93 | 0.2% | 2.0% | 0.96 | 0.67 | 1.0% | Bacteria; Proteobacteria; Gammaproteobacteria; Oceanospirillales; Oceanospirillaceae; Marinomonas |
| leaves | 397 | 0.0% | 1.7% | 0.85 | 1.44 | 0.9% | Bacteria; Proteobacteria; Alphaproteobacteria; Rhodobacterales; Rhodobacteraceae |
| leaves | 477 | 0.3% | 1.4% | 0.69 | 0.59 | 0.7% | Bacteria; Proteobacteria; Gammaproteobacteria; Alteromonadales; Alteromonadaceae; Simiduia |
| leaves | 123 | 0.0% | 1.4% | 0.68 | 0.90 | 0.7% | Bacteria; Proteobacteria; Gammaproteobacteria |
| leaves | 182 | 0.0% | 1.4% | 0.66 | 0.98 | 0.7% | Bacteria; Proteobacteria; Alphaproteobacteria; Rhodobacterales; Rhodobacteraceae |
| roots | 6 | 11.6% | 0.0% | 5.81 | 2.24 | 6.2% | Bacteria; Proteobacteria; Gammaproteobacteria |
| roots | 30 | 3.0% | 0.0% | 1.49 | 1.24 | 1.6% | Bacteria; Proteobacteria; Gammaproteobacteria |
| roots | 61 | 3.0% | 0.1% | 1.48 | 1.08 | 1.6% | Bacteria; Proteobacteria; Epsilonproteobacteria; Campylobacterales; Campylobacteraceae; Arcobacter |
| roots | 90 | 2.2% | 0.0% | 1.09 | 1.70 | 1.2% | Bacteria; Proteobacteria; Gammaproteobacteria; Incertae_Sedis; Incertae_Sedis; Sedimenticola |
| roots | 47 | 1.9% | 0.0% | 0.93 | 0.56 | 1.0% | Bacteria; Proteobacteria; Gammaproteobacteria; Vibrionales; Vibrionaceae; Vibrio |
| roots | 60 | 1.8% | 0.0% | 0.89 | 1.63 | 0.9% | Bacteria; Bacteroidetes; Bacteroidia; Bacteroidales; Marinilabiaceae |
| roots | 86 | 1.7% | 0.0% | 0.83 | 1.27 | 0.9% | Bacteria; Proteobacteria; Gammaproteobacteria; Incertae_Sedis; Incertae_Sedis; Sedimenticola |
| roots | 148 | 1.6% | 0.0% | 0.80 | 1.90 | 0.9% | Bacteria; Proteobacteria; Deltaproteobacteria; Desulfobacterales; Desulfobacteraceae; Desulfobacula |
| roots | 75 | 1.6% | 0.0% | 0.79 | 1.31 | 0.8% | Bacteria; Proteobacteria; Gammaproteobacteria |
| roots | 163 | 1.5% | 0.0% | 0.74 | 2.18 | 0.8% | Bacteria; Proteobacteria; Deltaproteobacteria; Desulfobacterales; Desulfobulbaceae |
| roots | 1079 | 1.4% | 0.3% | 0.70 | 0.63 | 0.7% | Bacteria; Proteobacteria; Betaproteobacteria; Methylophilales; Methylophilaceae; Methylotenera |
| roots | 43 | 1.3% | 0.2% | 0.67 | 0.48 | 0.7% | Bacteria; Proteobacteria; Gammaproteobacteria; Oceanospirillales; Oceanospirillaceae; Reinekea |
| roots | 119 | 1.3% | 0.0% | 0.65 | 0.97 | 0.7% | Bacteria; Proteobacteria; Deltaproteobacteria; Desulfobacterales; Desulfobacteraceae; Desulfobacula |
